# Treatment outcomes in classic Hodgkin lymphoma: 5‐year update from the Brazilian Hodgkin Lymphoma Registry

**DOI:** 10.1002/jha2.804

**Published:** 2023-10-04

**Authors:** Irene Biasoli, Nelson Castro, Carolina Colaço Villarim, Fabiola Traina, Carlos Sergio Chiattone, Monica Praxedes, Cristiana Solza, Leila Perobelli, Otavio Baiocchi, Rafael Gaiolla, Carla Boquimpani, Valeria Buccheri, Caroline Bonamin Sola, Roberta de Oliveira de Paula e Silva, Ana Carolina Ribas, Giovanna Steffenello, Katia Pagnano, Andrea Soares, Carmino de Souza, Nelson Spector

**Affiliations:** ^1^ School of Medicine Universidade Federal do Rio de Janeiro Rio de Janeiro Brazil; ^2^ Hospital de Cancer de Barretos Barretos São Paulo Brazil; ^3^ Liga Norte Rio‐Grandense contra o Cancer Rio Grande do Norte Brazil; ^4^ Department of Medical Imaging Haematology, and Oncology Ribeirão Preto Medical School University of São Paulo Ribeirão Preto São Paulo Brazil; ^5^ Sao Paulo Santa Casa Medical School São Paulo Brazil; ^6^ Universidade Federal Fluminense Niteroi Rio de Janeiro Brazil; ^7^ Universidade do Estado do Rio de Janeiro Rio de Janeiro Brazil; ^8^ Hospital Brigadeiro São Paulo Brazil; ^9^ UNIFESP São Paulo Brazil; ^10^ UNESP Botucatu São Paulo Brazil; ^11^ HEMORIO Rio de Janeiro Rio de Janeiro Brazil; ^12^ Instituto do Cancer do Estado de São Paulo/Hospital das Clinicas ‐ Faculdade de Medicina da Universidade de São Paulo São Paulo Brazil; ^13^ Universidade Federal do Parana Parana Brazil; ^14^ Universidade Federal de Minas Gerais Minas Gerais Brazil; ^15^ CEPON Santa Catarina Brazil; ^16^ Universidade Federal de Santa Catarina Santa Catarina Brazil; ^17^ Hematology and Hemotherapy Center University of Campinas Sao Paulo Brazil

**Keywords:** Hodgkin disease, Hodgkin lymphoma, outcome

1

In recent decades, significant advancements have occurred in the treatment of Hodgkin lymphoma (HL) [1]. However, limited data exist regarding HL in low‐ and middle‐income countries (LMIC) [2, 3]. The Brazilian Hodgkin Lymphoma Registry was established in 2009 and aims to collect comprehensive information in a prospective cohort of HL patients [4, 5, 6, 7]. In this updated analysis, we present data on patients diagnosed up to 2018, with a median follow‐up of 5 years.

There are currently 17 participating institutions, most being public university hospitals. All patients receive treatment within the Brazilian public health system, which provides free and universal healthcare. Further details of the registry are described in the Supporting Information.

From January 2009 to December 2018, 1507 patients with HL were included in the database. Forty‐seven patients with nodular lymphocyte‐predominant HL, 12 patients younger than 13 years, and 91 patients with HIV were excluded. Among the remaining 1357 patients, 28 were not included in the outcome analysis for several reasons, leaving 1329 patients with classic HL available for the analysis.

The median age of the patients was 30 years (range: 13–90 years). The median time from onset of symptoms to diagnosis was 6 (0–60) months. Patients' characteristics at diagnosis are shown in Table 1.

**TABLE 1:** Patients’ characteristics at diagnosis.

Characteristics	*N* = 1329 (%)
Female sex	660 (50)
PS ECOG > 1	212 (16)
Ann Arbor stage	
IA	40 (3)
IB	9 (0.5)
IIA	236 (18)
IIB	320 (24)
IIIA	63 (5)
IIIB	220 (17)
IVA	59 (4)
IVB	376 (28)
Missing	6 (0.5)
GHSG classification	
Limited	87 (7)
Intermediate	337 (25)
Advanced	862 (65)
Missing	43 (3)
Bulky mediastinal involvement	
Yes	384 (29)
No	941 (70)
Missing	4 (1)
Presence of B symptoms	929 (70)
Histopathology	
Nodular sclerosis	991 (75)
Mixed cellularity	175 (13)
Lymphocyte‐rich	18 (1)
Lymphocyte‐depleted	19 (1)
Classical HL unclassified	126 (10)
IPS	
0–2	781 (59)
>2	525 (40)
Missing	23 (1)

*Note*: Bulky mediastinal was defined by mediastinal mass more than one‐third of the transverse diameter of the thorax or mediastinal mass over 10 cm.

Abbreviations: ECOG, Eastern Cooperative Oncology Group; GHSG, German Hodgkin Study Group; IPS, International Prognostic Score; PS, performance status.

Only 24% (315) of patients have been staged using positron emission tomography–computed tomography (PET‐CT), and 60% (794) have been evaluated with an end‐of‐treatment PET‐CT scan. Despite the low use of PET‐CT, there was a substantial rise in its usage for both staging (11% vs. 36%, *p* < 0.0001) and end‐of‐treatment evaluations (40% vs. 79%, *p* < 0.0001) when comparing patients included during the periods of 2009–2014 and 2015–2018.

The median time from diagnosis to the beginning of treatment was 25 days (0–449 days). Most patients (1107/1329, 83%) received treatment within 2 months of diagnosis. ABVD (doxorubicin [adriamycin), bleomycin, vinblastine, dacarbazine) was the first‐line treatment in 1248 (94%) patients. Among those who received ABVD, patients with limited disease received a median of four cycles (2–6), patients with intermediate disease a median of four cycles (2–8), and patients with advanced disease a median of six cycles (1–8). The median duration of each cycle was 27 days. The remaining patients were treated with BEACOPP‐based regimens.

Radiotherapy (RT) was used in 72% of patients with limited disease, 59% with intermediate, and 28% with advanced disease. The median time from the end of chemotherapy to the beginning of RT was 1.7 months. The median dose of RT was 30 Gy for all risk groups. There was a decline in the use of RT (44% vs. 35%, *p* = 0.002) from 2009–2014 to 2015–2018. This decline was observed in localized disease (66% vs. 56%, *p* = 0.02) and in advanced disease (32% vs. 24%, *p* = 0.01). Also, there was an increase in the use of involved‐field RT (77% vs. 90%, *p* < 0.001), accompanied by a decrease in the use of extended fields comparing both periods.

Thirty‐four (2.6%) patients died during first‐line treatment. In 30 (88%), the cause of death was an infection or a complication of treatment (five deaths due to lung toxicity, two due to cardiotoxicity, and one due to gastrointestinal bleeding). Three patients died of disease progression and one of unknown causes. The mortality rates during treatment were 3.2% and 1.9% for the periods 2009–2014 and 2015–2018, respectively (*p* = 0.16). In addition, one patient died of cardiac failure (attributed to cardiotoxicity) 5 months after the end of treatment, and it was impossible to assess his treatment outcomes.

Among 1294 patients evaluated after completing front‐line treatment, the complete remission (CR) rate was 74% (961 patients), the unconfirmed CR rate was 7% (88 patients), the partial remission rate was 5% (60 patients), 2% (21 patients) had stable disease, and 12% (164 patients) had progressive disease.

Median follow‐up was 56 months (0–163 months) for all patients and 63 months (4.5–163 months) for alive patients. The 5‐year progression‐free survival (PFS) and 5‐year overall survival (OS) were 70% and 86%, respectively. The 5‐year PFS in limited, intermediate, and advanced diseases were 97%, 82%, and 62% (*p* < 0.0001). The 5‐year OS for limited, intermediate, and advanced disease were 100%, 94%, and 80% (*p* < 0.0001) (Figure 1).

**FIGURE 1:**
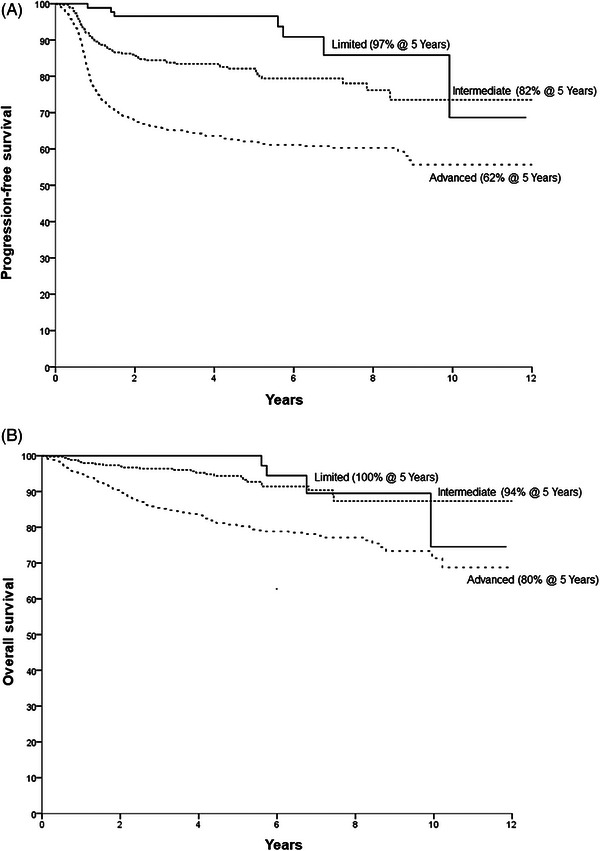
(A) Progression‐free survival according to German Hodgkin Study Group (GHSG) risk group classification. (B) Overall survival according to GHSG risk group classification.

In limited disease, there were no differences in the outcomes of patients treated with combined therapy or with chemotherapy only. In intermediate disease, patients who did not receive radiotherapy had inferior outcomes. Caution is required when interpreting these results, as non‐randomized physician decisions may introduce bias (see Supporting Information).

The impact of socioeconomic status (SES) on outcomes was analyzed in patients treated with ABVD. The 5‐year PFS in higher and lower SES were 75% and 60% (*p* < 0.0001). The 5‐year OS in higher and lower SES were 90% and 77% (*p* < 0.0001). The mortality rate during treatment was 5% and 1.1% for lower and higher SES (*p* < 0.0001). After adjustments for potential confounders, lower SES remained independently associated with poorer survival (hazard ratio [HR] 2.10 [1.52–2.90] for OS and HR 1.58 [1.26–1.99] for PFS).

The present analysis provides valuable insights about HL in Brazil. There was a predominance of advanced disease and high‐risk profile patients, which are key factors associated with inferior outcomes in patients with HL.

Treatment outcomes with ABVD in Brazil fall 10%–15% behind developed countries. For advanced disease, the 5‐year PFS was 62%, compared to approximately 75% in contemporary ABVD results [8]. This disparity stems from a poorer risk profile, and higher‐than‐expected mortality rates during treatment. Mortality rates associated with ABVD range from 0% to 0.3% in contemporary studies [9, 10, 11], while the rate was 2.6% in our patients. The main causes of treatment‐related deaths were infection and drug toxicities. Encouragingly, there is a recent trend of decreased mortality, potentially linked to reduced bleomycin use in older patients [12].

There has been a shift in therapeutic approaches. PET scan usage has increased, while the use of radiotherapy and larger fields has decreased. This suggests that new treatment recommendations are adopted more slowly in LMIC, possibly due to limited equipment and slow knowledge diffusion.

The influence of socioeconomic inequalities on cancer survival is well established, even in affluent nations [13, 14]. SES was an independent determinant of survival in the first analysis of the Brazilian HL Registry, and this finding was reaffirmed in this updated follow‐up [5]. However, the underlying mechanisms for this phenomenon remain elusive with the available data.

In conclusion, this study highlights the need to improve healthcare access and reduce treatment disparities in LMICs. Identifying and addressing factors that contribute to inferior results is essential for improving patient outcomes and overall healthcare equity.

## AUTHOR CONTRIBUTIONS

Irene Biasoli and Nelson Spector conceived and designed the study, analyzed and interpreted data, performed the statistical analysis, and wrote the manuscript. Nelson Castro, Carmino de Souza, Carolina Colaço Villarim, Fabiola Traina, Carlos Sergio Chiattone, Monica Praxedes, Cristiana Solza, Leila Perobelli, Otavio Baiocchi, Rafael Gaiolla, Carla Boquimpani, Valeria Buccheri, Caroline Bonamin Sola, Roberta Oliveira de Paula e Silva, Ana Carolina Ribas, Giovana Steffenello, Andrea Soares, and Irene Biasoli were the site investigators and/or responsible for collection and curation of study data. All authors collected data, critically reviewed, edited, and approved the article's final version.

## CONFLICT OF INTEREST STATEMENT

The authors declare they have no conflicts of interest.

## CLINICAL TRIAL REGISTRATION

The study is registered at https://www.clinicaltrials.gov/study/NCT02589548

## ETHICS STATEMENT

The ethics committees of the participating institutions approved the study in accordance with the ethical standards of the institutional and national research committees and with the 1964 Helsinki Declaration and its later amendments or comparable ethical standards.

## PATIENT CONSENT STATEMENT

All patients signed an informed consent.

## Supporting information

Supporting Information

## Data Availability

The corresponding author may provide the analyzed data supporting the study findings upon reasonable request, subject to data protection and applicable regulations, as the data are not publicly available due to ethical and privacy restrictions.
