# Supplementary material for: Treatment outcomes in classic Hodgkin lymphoma: 5‐year update from the Brazilian Hodgkin Lymphoma Registry
Source: EJHaem. 2023 Oct 4;4(4):1191–5. doi: 10.1002/jha2.804 (PMC10660107; doi:10.1002/jha2.804)
Supplement: Supplementary file 1 — Supporting Information [file JHA2-4-1191-s001.docx]

**Supporting Information**

**Patients and methods**

The Brazilian Prospective Hodgkin Lymphoma Registry contains information on clinical findings, treatment procedures, and patient outcomes. Socioeconomic status (SES) stratification was performed using an asset/education-based household index called "Criteria for Economic Classification”.^6^ Patients were categorized into higher (A1-C1) or lower (C2-E) SES groups. All treatment decisions were made at the physicians' discretion, according to each institution's clinical practice.

**Outcomes definitions**

Response to treatment was defined according to the 1999 international criteria.^7^ Imaging evaluation was based only on computed tomography (CT) scans because positron emission tomography (PET-CT) is still unavailable for many patients in Brazil. Progression was defined as relapse, failure to achieve complete remission (CR), or unconfirmed CR. Overall survival (OS) was calculated from the date of diagnosis to the date of death or last contact. Progression-free survival (PFS) was calculated from the date of diagnosis to the date of progression, death, or last contact. All patients were included at the date of diagnosis, which was the start of follow-up. Patients were followed until death or last contact. The mortality rate during treatment was defined as the proportion of patients who died of any cause during or up to 30 days after first-line treatment.

**Statistical analysis**

For crude association analysis, the chi-square or Fisher's exact test (two-sided) was used for categorical data, while the Mann-Whitney test was used for continuous data. Survival curves were estimated using the Kaplan-Meier method and compared using the log-rank test. The impact of SES on OS and PFS was evaluated in patients who received ABVD as first-line treatment. Cox regression analyses were used to assess the possible effects of SES on OS and PFS, controlling for a set of well-established prognostic factors that could be associated with SES. The covariates included in the model were: performance status (ECOG scale 0–1 or ≥ 2), IPS score (low-risk or high-risk), GHSG risk group classification (non-advanced or advanced), the time elapsed from the beginning of symptoms to diagnosis (≤ 4 or > 4 months) and histopathology (nodular sclerosis or non-nodular sclerosis). The results of the multivariate analyses were presented as hazard ratio (HR) of death or progression according to SES categories, with respective 95% confidence intervals (CI). The SPSS software (Chicago, IL, USA) was used for data analysis.

**Patients excluded from the outcome analysis**

Among the remaining 1357 patients, 28 were not included in the outcome analysis for the following reasons: 18 abandoned during first-line treatment, 6 did not receive any treatment (the diagnosis was established after they died), 3 received palliative treatments due to prohibiting co-morbidities and 1 received a diagnosis of another cancer and treatment was interrupted, leaving 1329 patients with classic HL available for the analysis. The age cut-off was established based on referral patterns and center-specific policies of the participating institutions.

**The role of radiotherapy in limited and intermediate disease**

Among patients treated with ABVD, there were 84 patients with limited disease, of which 23 were treated with chemotherapy (CT) only and 61 were treated with CT and RT. The median number of ABVD cycles was five for those treated with CT only, and four for those who received combined treatment. There were no differences in outcomes according to whether or not RT was given.

Among the 326 patients with intermediate disease, 134 patients were treated with CT only, while 192 patients received combined treatment. The median number of ABVD cycles was six for those treated with CT alone, and four for those who received with combined treatment. Inferior outcomes were observed among those who did not receive RT, with a 5-year PFS of 65% compared to 95% (P<0.0001) and a 5-year OS rate of 88% compared to 99%, P<0.0001). These results should be interpreted with caution since the treatment options were not randomized and treatment decisions were made at the physicians' discretion, according to each institution's clinical practice, and therefore potentially biased by hidden characteristics related to patients and treatment facilities.
